# Nematode and Strepsipteran Parasitism in Bait-Trapped and Hand-Collected Hornets (Hymenoptera, Vespidae, *Vespa*)

**DOI:** 10.3390/insects14040398

**Published:** 2023-04-20

**Authors:** Natsumi Kanzaki, Shun’ichi Makino, Hajime Kosaka, Katsuhiko Sayama, Keiko Hamaguchi, Shinji Narayama

**Affiliations:** 1 Kansai Research Center, Forestry and Forest Products Research Institute (FFPRI), 68 Nagaikyutaroh, Momoyama, Fushimi, Kyoto 612-0855, Japan; hamaguti@ffpri.affrc.go.jp (K.H.); narayama@ffpri.affrc.go.jp (S.N.); 2 Department of Forest Entomology, Forestry and Forest Products Research Institute, 1 Matsunosato, Tsukuba, Ibaraki 305-8687, Japan; makino@ffpri.affrc.go.jp; 3 Department of Mushroom Science and Forest Microbiology, FFPRI, 1 Matsunosato, Tsukuba, Ibaraki 305-8687, Japan; hkosaka@ffpri.affrc.go.jp; 4 Kyushu Research Center, Forestry and Forest Products Research Institute, 4-11-16 Kurokami, Chuo, Kumamoto 860-0862, Japan

**Keywords:** behavior manipulation, parasitism, *Sphaerularia*, *Vespa*, *Xenos*

## Abstract

**Simple Summary:**

Parasites sometimes manipulate host behavior to effectively utilize their hosts. The parasitism of host-manipulating parasites of hornets (*Vespa* spp.) was examined for baited or hand-collected hosts. There, two groups of endoparasites that sterilize their hosts, a nematode species, *Sphaerularia vespae*, and two species of parasitic insects, *Xenos* spp., were recovered during the survey. Although the nematode parasitism was scarcely observed (4 out of 1641 host hornets), the parasitism of *Xenos* spp. varied among host species between 0 and 17%. Interestingly, the rate of hornets parasitized by *Xenos* spp. was higher in baited individuals than in hand-collected ones. Because the bait trap attracts feeding (not foraging for colony) individuals, the parasitism manipulates the host to visit a feeding site, and the result is in good accordance with previous observations; i.e., parasitized hosts frequently visit and stay at feeding sites instead of foraging and reproducing. The present study could be the first case to quantify the *Vespa* host manipulation of *Xenos* spp. Genetically, both the *Xenos* spp. had four haplotypes in the mitochondrial cytochrome oxidase subunit I gene, and these were close to Japanese and some other East Asian genotypes.

**Abstract:**

The parasitism of two groups of host-manipulating parasites of hornets was examined in Kyoto, Japan. *Vespa mandarinia* (661 individuals), *V. simillima* (303), *V. analis* (457), *V. ducalis* (158), *V. crabro* (57), and *V. dybowskii* (4) were collected either by bait trap or hand collection with an insect net, and examined for their parasites. An endoparasitic nematode, *Sphaerularia vespae* was isolated from three overwintered gynes of *V. mandarinia* and a gyne of *V. ducalis*. While endoparasitic insects, *Xenos* spp., were recovered from 13 *V. mandarinia*, 77 *V. analis*, two *V. ducalis,* and three *V. crabro*, and those recovered from *V. analis* and others were molecularly identified as *X. oxyodontes* and *X. moutoni*, respectively. Comparing *Xenos* parasitism level and capturing methods, the parasitism level was significantly higher in trapped hosts than in hand-collected ones, suggesting that stylopized hosts are more strongly attracted to the food source (bait trap) compared with unparasitized hosts. The genotypes of *S. vespae* were identical to each other, and near identical to its type population. While each of the two *Xenos* spp. showed four mitochondrial DNA haplotypes. A phylogenetic comparison suggested that *Xenos* haplotypes found in the present study are close to those previously reported from Japan and other Asian countries.

## 1. Introduction

Hornets, or the eusocial wasps of the genus *Vespa*, have great importance to humans not only as natural enemies of agricultural and sanitary pest insects [1], but also as dangerous stinging agents sometimes causing casualties [2]. In addition, several *Vespa* species are important invasive species, such as *V. velutina* Lepeletier, 1836, and *V. mandarinia* Smith, 1852, of Asian origins, expanding their distributions in Europe [3] and North America [4], respectively. Therefore, considering their status as a group of social insects, and practical importance as beneficial and pest insects, the genus can be regarded as an interesting group of insects from many aspects [1].

As are other insect groups [5], *Vespa* wasps are associated with several different groups of parasites [1,6,7]. Some parasites manipulate their host’s behavior for their reproduction, and host manipulation by parasites has been intensively studied from ecological and evolutionary viewpoints [8]. If the hosts are serious pests, the parasites become important potential biocontrol agents.

Two groups of parasites of *Vespa* spp., i.e., nematodes in the genus *Sphaerularia* (Nematoda: Sphaerularioidea) and parasitic insects in the genus *Xenos* (Strepsiptera, Stylopidae), have been reported to manipulate the wasps’ behavior to complete their life cycle. *Sphaerularia vespae* Kanzaki, Kosaka, Sayama, Takahashi, and Makino, 2007, was described from *V. simillima* Smith, 1868, in Hokkaido, Japan [9]. The life cycle of the nematode has been examined by Sayama et al. [10]. The inseminated infective (free-living) females infest the body cavity (hemocoel) of hibernating gynes in their hibernating sites, e.g., inside the rotten wood, and develop into the parasitic female called ‘uterium’, which is the hypertrophied female reproductive system specialized to oviposition. Thereafter, the nematode eggs hatch in the host’s body cavity, and develop into juveniles taking nutrients from the host’s body. The parasitized gynes are sterilized, i.e., they cannot establish a colony, and fly around the feeding sites and potential hibernating sites for their own feeding and inoculate the nematode juveniles at the hibernating sites for the next generation gynes. The inoculated nematode juveniles develop into male and female adults, and mate at the hibernation site. Thereafter, the males die out there, and the inseminated females parasitize the overwintering gynes. Thus, the nematode manipulates the hosts to disperse their juveniles in potential hibernating sites [10] in a similar way as reported for its congener *S. bombi* Dufour, 1837, which parasitizes *Bombus* spp. [11,12]. In addition to these two nominal parasite species, an undescribed *Sphaerularia* species has been reported from paper wasps, *Parapolybia* spp. [13]

The strepsipteran parasites, *Xenos* spp., parasitize hornets and paper wasps [1,13,14,15,16,17,18,19,20]. The life cycle and the host manipulation of *Xenos* spp. have been illustrated by several authors [1,13,14,15,16,17,18,19,20]. The parasite shows remarkable sexual dimorphism, i.e., winged male and magot-like neotenic female: the female stays in the host’s body, and the male flies for mating. The host hornets (workers or gynes) parasitized by an inseminated female parasite(s) overwinter like unparasitized gynes, and frequently visit and stay at feeding sites to release the first instar infective larvae (triangulin) of the parasites at their feeding sites, e.g., sap flow and flower honey, in spring. The triangulin larvae probably sneak into nests of the hosts by attaching to adult hornets, and invade host larvae in the nests. Infected (“stylopized”) hosts, even though emerging as adults, do not conduct normal behavior as workers or reproductives, but release the triangulins of the parasites at their feeding sites. This infection cycle could be repeated once or more during the active season of the host wasps/hornets. Because the nematode infection occurs during hibernation, stylopized gynes could be infested by the nematode. However, so far, their co-parasitism has not been reported.

Therefore, both groups of parasites potentially affect *Vespa* populations by manipulating the wasps. However, the basic ecological information for these parasites is still limited. In this study, we investigated the distribution and host range of these parasites in the Kyoto area, in the middle of Japan, where records on parasitism are sparse, to better understand the prevalence of the parasites, and make detailed molecular analyses, e.g., genomic and transcriptomic analyses of the host–parasite interactions. During the collections, several new findings emerged on the capturing and parasitism patterns of wasps, which are reported herein.

## 2. Materials and Methods

### 2.1. Collection and Dissection of Wasps

The hornets were collected with two different methods, bait traps and hand collection. Seven traps with liquid bait (sweet potato liquor: orange juice = 1:1) [16] were placed at a height of ca. 1.2 m above ground in the experimental stands of the Kansai Research Center, Forestry and Forest Products Research Institute, Kyoto, Japan, at the end of March 2021, before the wasps’ active season, to the middle December, after their active season. During this collection, bait was replaced approximately every 10 days. The wasps captured by the bait trap were collected almost daily, 5–7 times a week. Hand collection was performed during the same period with the bait traps in the collection area where the traps were installed. Hornets were collected with an insect net 5–7 times a week while they were feeding or flying where one or two collector(s) walked throughout the collection site, and collected the wasps using an insect net with a handle of maximum 4.0 m long. Feeding individuals were sitting on tree trunks (for tree sap) of mostly *Quercus* spp. or on flowers (nectar) of *Cayratia japonica* (Thunb., 1784), *Dendropanax trifidus* (Thunb., 1784) and unidentified herbs; flying individuals were often holding prey, or flying in open spaces, around flower or tree trunks without carrying prey. Thus, ‘flying’ may include some hunting and feeding individuals.

Collected wasps were morphologically identified for species, caste, and sex, stored in refrigerator, and dissected under a stereomicroscope (SMZ1500, Nikon, Tokyo, Japan) within a day after the collection. The queen and worker were distinguished during the dissection by the body size and the development of ovaries. Because the parasites are supposed to be in the host abdomen, the abdomen of the wasps was taken and cut open with a pair of tweezers or dissecting scissors to examine the presence of their parasites. The dissected wasps were individually vouchered in a glass vial filled with 99.5% ethanol in the laboratory. When found, nematodes and strepsipteran parasites were stored in a glass vial filled with 99.5% ethanol for further molecular identification, and the number and sex of strepsipteran parasites were recorded for each host. In addition, some nematode materials were killed by heat (55 °C for 30 s), and fixed in TAF (2% triethnolamine, 3.5% formalin) as morphological vouchers. The number, caste, and parasitism level of wasps were compared among species, and between the two collection methods. The statistical analyses were conducted using the Chi-square test.

### 2.2. Identification of Parasites

When parasites were found from the host wasps, they were molecularly identified. Approximately 4 kb of ribosomal DNA (rDNA) including near-full length of the small subunit (SSU), internal transcribed spacer region (ITS), and D1–D4 extension segments of the large subunit (D1–D4 LSU) was determined for nematodes, and ca. 0.6 kb of partial code of mitochondrial cytochrome oxidase (mtCOI) was determined for the strepsipteran parasites.

Several (four to six) parasitic juveniles of the nematode were digested using nematode digestion buffer [21,22] as the template DNA, and the rDNA sequence was determined with PCR-direct (Sanger) sequencing using several first PCR and internal primers [23] (Supplementary Table S1). The sequence analysis was conducted for all parasitized hosts.

For the strepsipteran parasites, only females were examined for their sequences because almost all male parasites were already emerged (dispatched from host body), and their cocoons retained in the host body were tangled with host tissue. A small piece (less than 10 μL in volume) of parasite body tissue was taken with a pair of tweezers, transferred to 100 μL of DNA extraction solution (PrepMan Ultra Sample Preparation Reagent, ThermoFisher, Waltham, MA, USA), and kept at 95 °C for 10 min. The supernatant was used as PCR template, and the mtCOI of each sample was amplified and sequenced using a universal primer set (Supplementary Table S1).

Determined sequences were then compared with those deposited in the GenBank database, https://blast.ncbi.nlm.nih.gov/Blast.cgi?PROGRAM=blastn&PAGE_TYPE=BlastSearch&LINK_LOC=blasthome (accessed on 4 February 2022) using BLAST program, https://blast.ncbi.nlm.nih.gov/Blast.cgi?PROGRAM=blastn&PAGE_TYPE=BlastSearch&LINK_LOC=blasthome (accessed on 4 February 2022) for their species identification. In addition, their intraspecific genotypic diversity was examined using the program BioEdit [24], where the conspecific sequences were aligned, and the variations were examined manually. Thereafter, to compare *Xenos* spp. sequenced obtained in the present study and previously reported ones, an unrooted phylogenetic tree was generated using MrBayes 3.2 software [25,26] with a substitution model (GTR + G) selected using MEGA X software [27], where four chains were run for 4 × 10^6^ generations, and the Markov chains were sampled at intervals of 100 generations [28]. Two independent runs were performed, and after confirming convergence and discarding the first 2 × 10^6^ generations as burn-in, the remaining topologies were used to generate a 50% majority-rule consensus tree.

## 3. Results

In addition to the following data, row data for collection are provided as a spreadsheet (Supplementary Data S1).

### 3.1. Hornet Captures

**Figure 1:**
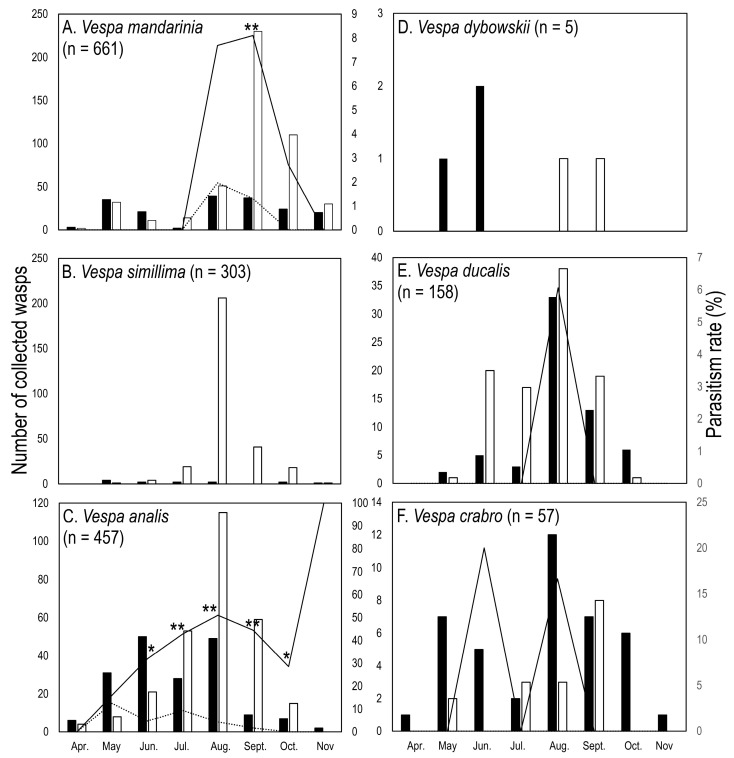
Number of collected wasps and rate of *Xenos* parasitism: (**A**): *Vespa mandarinia*; (**B**): *V. simillima*; (**C**): *V. analis*; (**D**): *V. dybowskii*; (**E**): *V. ducalis*; (**F**): *V. crabro*. Number of collected wasps (bar graph) and the parasitism rate (line graph) of each month are shown on left and right axis, respectively. Black and white bars are the numbers of trapped and hand-collected wasps, respectively, and solid and dotted line graphs indicate the parasitism ratio of trapped and hand-collected wasps, respectively. Asterisks on the bar graph indicate that the rate is significantly different between trapped and hand-collected wasps (*: *p* < 0.05; **: *p* < 0.01). Parasitism was not observed in *V. simillima* and *V. dybowskii*.

In total, 1641 wasps of six *Vespa* species were captured during April and November (Figure 1, Table 1, Supplementary Data S1). The most abundant species was *V. mandarinia* (661 individuals), followed by *V. analis* Fabricius,1775 (457), *V. simillima* (303), *V. ducalis* Smith, 1858 (158), and *V. crabro* Linnaeus 1758 (57). A social parasite, *V. dybowskii* André, 1884, was rare, and only five individuals were captured during the collection season (Figure 1, Table 1, Supplementary Data S1). Thus, *V. dybowskii* is excluded from the following analysis.

The capturing pattern varied among the species. Although all six species were captured by both collection methods, the majority of the dominant four species (*V. mandarinia*, *V. analis*, *V. simillima,* and *V. ducalis*) were captured with an insect net, and this tendency was conspicuous in *V. simillima*, where 290 individuals out of 303 total individuals were obtained by hand collection. Although detailed location data were not taken, the species was mostly collected from flowering trees. Contrastingly, *V. crabro* was collected more by bait trapping (41 out of 57 individuals).

*Vespa mandarinia*, *V. analis*, *V. simillima,* and *V. crabro* were collected throughout the collection season (April to November), but *V. ducalis,* which exclusively preys on larvae and pupae of paper wasps [1], was only collected from May to October; i.e., the active season of the species was shorter than the other four species because of their prey preference.

The rate of *Xenos* parasitism was different between collection methods; i.e., the rate was generally higher in bait-trapped hornets than for hand-collected ones. *Xenos oxyodontes* Nakase and Kato, 2013, exclusively obtained from *V. analis* parasitized 36.3% and 4.7% of bait-trapped and hand-collected hosts, respectively (Figure 1, Table 1, Supplementary Data S1) (*p* < 0.01). In addition, although the differences were not statistically significant, *X. moutoni* (du Buysson, 1903) parasitized 3.3% and 1.5% of bait-trapped and hand-collected *V. mandarinia*, respectively; the parasites were found only from trapped individuals in *V. ducalis* and *V. crabro* (Figure 1, Table 1, Supplementary Data S1).

### 3.2. Nematode Parasitism

Parasitic nematodes were found from four (three *V. mandarinia* and a *V. ducalis*) queens in May (Table 2). The nematodes were all identified as *Sphaerularia vespae* based on molecular sequences; i.e., the SSU, ITS, and D2-D3 LSU of the nematodes were identical to each other, and to the type material of the species (SSU + ITS: AB300595; D2–D3 LSU: AB300596).

### 3.3. Strepsipteran Parasitism

Strepsipteran parasites (*Xenos* spp.) were recovered from four species (*V. mandarinia*, *V. analis*, *V. ducalis,* and *V. crabro*) with various parasitism rates (Figure 1, Table 1, Supplementary Data S1). The total rate was variable among four species (Chi-square test, *p* < 0.01), and the rate was significantly higher in *V. analis* (17.2%) than in *V. mandarinia* (2.0%) and *V. ducalis* (1.3%) (*p* < 0.01), but the difference is not significant between *V. analis* and *V. crabro* (5.3%) (*p* = 0.062) and other combinations (*p* > 0.6) (Holm’s multiple comparison test).

**Figure 2:**
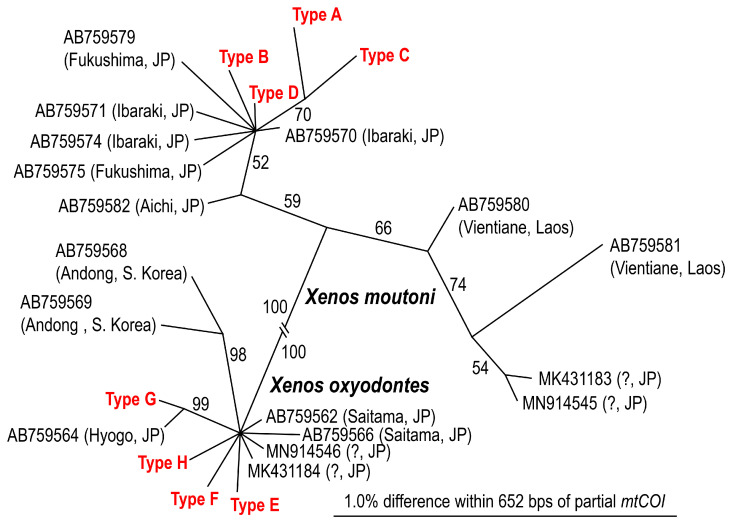
Phylogenetic relationship among haplotypes of two *Xenos* spp. Unrooted tree was generated with Bayesian analysis under GTR + G model (lnL = −1244.934; freqA = 0.33, freqC = 0.15, freqG = 0.10, freqT = 0.42; R(a) = 0.93, R(b) = 8.67, R(c) = 1.45, R(d) = 0.48, R(e) = 11.29, R(f) = 1.00; Pinva = n/a; Shape = 0.07). Posterior probability values exceeding 50% are given on appropriate clades.

Based on mtCOI sequence analysis, the parasites were identified as two different species, *X. moutoni* parasitizing *V. mandarinia*, *V. ducalis,* and *V. crabro*, and *X. oxyodontes,* which was exclusively recovered from *V. analis* (Figure 2; Table 3). Both species showed intraspecific variation in four genotypes (haplotypes) for each species (Figure 2; Table 3). The phylogenetic analysis suggested that these two species are clearly separate, and genotypes found in the present study are included in each species’ clade, and close to other Japanese isolates (Figure 2).

The sex ratio (percentage of female) of *Xenos* spp. varied between parasite species and among host wasps. The ratio was more female-biased in *X. moutoni* (90%), where only two males were found from *V. crabro*, and only female parasites were found from *V. mandarinia* and *V. ducalis*. The number of parasites was mostly one for these three species (Table 4 and Table 5, Supplementary Data S1). Contrastingly, the sex ratio in *X. oxyodontes* was approximately equal (54.3%), and the number of parasites per host varied from one to nine (Table 4). The sex ratio of the species was female-biased in the early season, and the ratio gradually decreased toward the late season (Table 5, Supplementary Data S1).

## 4. Discussion

Several different types of data were presented in this study, and some are dependent on each other. Therefore, these data will be discussed separately as a general capturing pattern in relation to capturing method in Section 4.1 and Section 4.2, nematode parasitism in Section 4.3, the biology of *Xenos* spp. in Section 4.4 and Section 4.5, and genetic diversity of *Xenos* spp. in the research site in Section 4.6, followed by additional remarks in Section 4.7.

### 4.1. Capture Outcomes of Bait-Trapping Versus Hand-Collecting Hornets

Out of the six hornet species collected, more individuals were obtained by hand-collection than bait traps in four hornet species (*V. mandarinia*, *V. simillima*, *V. analis,* and *V. ducalis*) (Figure 1, Table 1, Supplementary Data S1). While *V. crabro* was collected more by bait traps (Figure 1, Table 1, Supplementary Data S1). This difference in the capturing pattern probably reflects the intensiveness of hand-collection and behavioral characteristics of hornet species. Hand collection could have covered a wider area than the traps, because the traps were settled on the trees, and the hornets that visited these trees were collected, but the hand-collection was conducted through all areas including the tree stands. While the hand collection was conducted only during the daytime, the *V. crabro* exhibits foraging and feeding activity in the early morning and late evening into the night as well, i.e., the periods when the hand-collection cannot cover [29].

Interestingly, 95.7% (290 out of 303) of *V. simillima* were hand-collected, and only 13 individuals (4.3%) were bait-trapped. Based on previous bait trap-based surveys [16,30,31,32] and their own survey, Kudô et al. [33] suggested that *V. simillima* is dominantly collected by bait traps in cooler regions, but not in warm areas, which our trapping study supports; i.e., *V. simillima* was not trapped in Kyoto, which is in a relatively warm temperate zone. However, a fair number of *V. simillima* were captured by hand mostly from flowering trees, suggesting that although the species was commonly distributed in the collection site, the traps failed to attract it. The bait traps used in the present and previous studies in Japan mainly contain liquor and/or fruit juice [16,17,31,32,33], which emit odors similar to fermented tree sap or fruits. Thus, *V. simillima* possibly prefers other food sources, e.g., flower honey, in warmer regions. Otherwise, considering the body size, i.e., *V. simillima* is the smallest *Vespa* species in Japan, *V. simillima* might be outcompeted from tree sap by the larger *Vespa* spp., and utilize other available resources, i.e., flower honey. This difference in the capturing pattern suggests that multiple types of traps are necessary to monitor the *Vespa* fauna in warmer regions [34].

### 4.2. Comparison of Collection Methods

In all *Vespa* species where *Xenos* occurred, the parasitism level was consistently higher in the bait-trapped individuals than in hand-collected ones (Figure 1, Table 1, Supplementary Data S1). This was shown in the most marked way by *V. analis* in which the level was significantly higher in bait-trapped wasps than in hand-collected ones both in total number and monthly collections during June to October (Figure 1, Table 1, Supplementary Data S1).

In the present study, trapped hosts are considered to be attracted to odors similar to tree sap, which seems to account for the higher levels of *Xenos* parasitism in bait-collected as compared to hand-collected individuals. Further, almost all parasitized hornets in the hand-collected samples were obtained while they were feeding at or near tree sap holes. These results are consistent with the assumption that *Vespa*-associated *Xenos* spp. manipulate host adults by making them frequent feeding sites such as tree sap holes where the parasitic females may release the first-instar larvae [1,16].

A similar, or basically the same, behavioral modification has been reported in a paper wasp, *Polistes dominula* (Christ, 1791)—*Xenos vesparum* Rossius, 1793 system [19]. There, the parasitized host desists from the colony task and aggregates their own food source, trumpet creeper bushes, to feed and release infective larvae of the parasite [19]. This behavioral modification could be a common characteristic of these *Xenos* species.

### 4.3. Nematode Parasite

*Sphaerularia vespae* was collected from two host species, three queens of *V. mandarinia* and a queen of *V. ducalis* (Table 1, Supplementary Data S1). The nematode was originally recorded from *V. simillima* in Hokkaido, Japan [9,35], and subsequently found from other *Vespa* spp. from various areas in Japan [36]. Sayama et al. [35] reported that the parasitism rate of the nematode was very high in *V. simillima* from the Hokkaido region; i.e., more than 60% of overwintered gynes (new queens) collected with bait traps were infected by the nematode in some populations. However, in the present study, the nematode was found only from *V. mandarinia* and *V. ducalis*, and the parasitism rate was much lower, 1.6% (three out of 182) and 2.0% (one out of 50) of overwintered gynes, respectively, and none of nine overwintered gynes of *V. simillima* was infested by the nematode (Supplementary Data S1). We do not have a clear explanation for this difference in the infection rate between the Hokkaido and Kyoto populations, because the data for the infection rate have not been examined in different localities. The nematodes are disseminated to the hosts’ hibernation site because of the parasitized hosts’ aberrant behavior, and infest new hosts during hibernation [10]. Therefore, the difference in environmental conditions, i.e., subarctic (Hokkaido) and temperate (Kyoto) zones, could affect wasps’ behavior, e.g., segregation of hibernation sites between *V. simillima* and others as observed in feeding preference.

### 4.4. Levels of Xenos Parasitism in Bait-Trapped Hosts

The parasitism rate varied among host species, especially in trapped individuals; i.e., 36.3% (66 out of 182) of *V. analis* were parasitized by *X. oxyodontes*; 3.3% (6/181) of *V. mandarinia*, 3.2% (2/62) of *V. ducalis,* and 7.3% (3/41) of *V. crabro* were parasitized by *X. moutoni*, and no parasitism was found in *V. simillima* (Table 1, Supplementary Data S1).

The *Xenos* parasitism rate of *Vespa* spp. has been examined in several previous studies using bait traps (Table 6). Makino and Yamashita [16] collected the wasps in Miyazaki, Japan, and reported that four species (3457 individuals) of the wasps were captured, and the parasitism rate was high (10.6%) in *V. analis*, followed by *V. mandarinia* (3.8%), *V. ducalis* (3.8%), and *V. simillima* (1.4%). Subsequently, Makino [30] reported a similar pattern based on three seasons of trapping in Ibaraki, Japan; i.e., the parasitism rate was higher in *V. analis* (15–20%) than that in *V. simillima*, *V. ducalis,* and *V. crabro* (less than 2%), and no parasitism was observed in *V. mandarinia*. Similarly, Sayama [17] examined the parasitism rate for 10 years in Hokkaido, Japan, and collected five species of *Vespa* spp. There, approximately 8% of *V. analis* and less than 0.5% of *V. simillima* and *V. crabro* were stylopized, but no parasitism was confirmed in *V. mandarinia* and *V. dybowskii* (only one case of *Xenos* parasitism in *V. dybowskii* was observed in a worker of a collected nest). On the other hand, Oyaizu and Kudô [32] reported that the rate was rather high in *V. mandarinia* (16.7–23.5%), and *V. analis* (8.8–16.7%) was not clearly different from those of *V. ducalis* (12.5%) and *V. crabro* (10.1%), but higher than that of *V. simillima* (0.4–1.2%), based on two years of collections in Niigata, Japan.

Although the levels of parasitism not only vary among the host species but among the studies, the level is consistently high (ca 8.0–36.3%) in *V. analis*; a low level of parasitism has been found in *V. ducalis* (3.2–12.5%) and *V. crabro* (<0.5 to 10.1%); the level of parasitism is consistently low in *V. simillima* (<2.0%); and the level is highly variable in *V. mandarinia* (0–23.5%) in bait-trapped hosts (Table 6) [16,17,30,32]. This suggests that the affinity between *V analis* and *X. oxyodontes* is higher than that between the other *Vespa* spp. and *X. moutoni*, and that *V. simillima,* which has two strepsipteran parasite species [37], has a low affinity with both parasite species. Our results are in good accordance with the previous studies for high and low parasitism rates in *V. analis* and *V. simillima*, respectively, and generally similar to the pattern in Miyazaki. Although the number of examined localities was not sufficient, both Kyoto and Miyazaki belong to the relatively warm temperate zone, and thus, environmental conditions could affect the pattern of parasitism via the wasps’ behavioral pattern. For example, *V. simillima* was mostly collected by hand from the flower honey; i.e., the feeding (and possibly hunting) site of *V. simillima* is segregated from other hornet species in the experimental site, and the segregation allowed *V. simillima* to avoid the parasites derived from the other *Vespa* spp.

### 4.5. Number and Sex Ratio of Xenos *spp.*

The number of *Xenos* parasites per host varied between one to nine, and in most cases, one male or female was recovered from a parasitized host. A single female of *X. moutoni* mostly parasitized *V. mandarinia*, *V. ducalis,* and *V. crabro* (Table 4, Supplementary Data S1). While the number varied in *X. oxyodontes*, five females and four males were found in an individual host at maximum (Table 4, Supplementary Data S1).

Oyaizu and Kudô [32] reported the number of *Xenos* spp. per host varied from one to two, which is in good accordance with our data for *X. moutoni*, but multiple parasitisms of *X. oxyodontes* were not observed in the study. While, Makino and Yamashita [16] reported that the number varied from one to four *X. oxyodontes* in *V. analis* one to five *X. moutoni* in the other species, and 22 out of 102 (22%) of *V. analis* and 16 out of 84 (19%) of other *Vespa* spp. (*V. mandarinia* and *V. ducalis*) were parasitized by multiple individuals of *Xenos* spp.; i.e., the rate of multiple parasitisms is similar between two *Xenos* spp. In the present study, multiple parasitisms were observed in 17 out of 77 (22.1%) *V. analis* and none of the 17 others; i.e., the rate of multiple parasitisms is similar in *X. oxyodontes*, but the rate is lower in *X. moutoni*. The rate could be affected by the population density, behavior, and interspecific interaction of host wasps, but further investigation is necessary to explain these differences.

The overall sex ratio of *Xenos* individuals on the host throughout the season was greatly different between the two species, i.e., strongly female-biased in *X. moutoni* (female ratio = 90% in total), and mostly even (54.3%) in *X. oxyodontes* (Table 5, Supplementary Data S1). The female ratio of *X. oxyodontes* gradually decreased according to the season; i.e., only females that overwintered with host wasps were found in the early season (April and May), and the female ratio decreased to zero by the end of collections (Table 5, Supplementary Data S1). The pattern in *X. oxyodontes* is in accordance with the observation by Makino and Yamashita [16] and Makino [30]. There, although the *Xenos* species was all regarded to be *X. moutoni*, because *X. oxyodontes* was not separated at the time, males appear in the late season (mid-August) in Miyazaki [16], and *X. oxyodontes* males appear in July in Ibaraki [30]. Matsuura and Yamane [1] suggested that female parasites overwinter parasitizing the host, and reproduce three or more generations during the host’s active season. While although the parasite species is not specified, Sayama [17] considered that the *Xenos* parasite is univoltine in Hokkaido. The data in the present study, i.e., the female ratio decreased to zero more or less linearly, suggest that the parasite is univoltine at the study site. There, female parasites are collected from overwintered hosts in the early season, and next-generation parasites showing an even sex ratio appear in the middle season. Thereafter, host hornets parasitized by female parasites hibernate earlier than a regular queen, and the hosts harboring the male parasite cocoon can be captured in the late season. Although the number of generations (univoltine or bivoltine; i.e., the parasite could have reproduced two generations in the middle season) is not clearly examined, the number could be affected by the environment, especially the temperature, because the observation by Matsuura and Yamane [1] (possibly three or more generations) and Sayama [17] (one generation) were yielded from cold and warm areas, respectively. Moreover, a similar bivoltine cycle has been confirmed in *Polistes dominula* paper wasp and its parasite, *Xenos vesparum* based on field observations [19]. To confirm the generation time of *Vespa*-associated *Xenos* spp. in relation to environmental conditions, further field observations in the different areas are necessary.

We do not have a clear explanation for the female-biased sex ratio in *X. moutoni*; although, this could be simply because of the small (insufficient) number of collected materials. Because *X. oxyodontes* was separated from *X. moutoni* recently [37], two *Xenos* spp. were not distinguished in the previous studies. Therefore, the difference in the sex ratio is not determined whether it is derived from genetic differences related to the species-specific reproductive strategy or phenotypic variation in the species; e.g., the sex ratio of parasites is often variable among conspecific populations, and sometimes among subpopulations depending on the population density [38,39]. To understand the seasonal pattern of sex ratio in each *Xenos* spp. re-examination of previous data and further collections with species identification is necessary. We also need *Xenos* sex ratios in hornet nests, from which stylopized adults disperse into the field.

### 4.6. Molecular Identification and Genotypes of Xenos *spp.*

Two *Xenos* spp. were obtained from four host species, i.e., *X. moutoni* from *V. mandarinia*, *V. ducalis,* and *V. crabro*, and *X. oxyodontes* from *V. analis*.

*Xenos oxyodontes* was recently separated from *X. moutoni* based on molecular and morphological characters by Nakase and Kato [37]. Nakase and Kato [37] examined host-parasite associations of the two *Xenos* spp. and concluded that the two species segregate their hosts: *X. moutoni* is parasitic on *V. mandarinia*, *V. ducalis*, *V. crabro*, and *V. simillima*, while *X. oxyodontes* mainly parasitizes *V. analis*, and much less frequently *V. simillima*. The isolation pattern of *Xenos* spp. in the present study corroborates the observations of Nakase and Kato [37]; i.e., *X. oxyodontes* was exclusively found from *V. analis*, and those obtained from the other host species were all *X. moutoni* (Table 2 and Table 3; Supplementary Data S1).

Both *Xenos* spp. showed intraspecific variations in mtCOI sequences, and four haplotypes were recognized within each species (Figure 2; Table 3), suggesting that multiple (at least four) female lines are present in the collection site. The sequences of these haplotypes were phylogenetically compared with those deposited in the GenBank database, and visualized as an unrooted tree (Figure 2). Four haplotypes of *X. moutoni* (Types A–D) belonged to a phylogenetic clade including other conspecific Japanese materials; although, the posterior probability (PP) support was not high (59%) (Figure 2). *X. oxyodontes* haplotypes found in Kyoto were close to the other Japanese materials, and two Korean isolates were separated from them with relatively high (98%) PP support (Figure 2). However, in both species, a clear phylogeographical pattern was not recognized among Japanese materials, possibly because the sequence variation was relatively low, with only four variable sites within 652 bps in the specimens collected in the present study (Table 3). In *X. moutoni*, three haplotypes (A–C) were found only once, and all were isolated from *V. mandarinia*, and the dominant haplotype (D) from 13 individuals was found from three wasp species (Table 3A). The relationship between haplotype and host species is not clear because the number of sampled specimens was not sufficient.

### 4.7. Additional Remarks

In the present study, two groups of host-manipulating parasites of hornets were examined using two different collection methods, bait trap and hand-collection, mostly focusing on *Xenos* spp. and the capturing pattern was in good accordance with the manipulation of *Xenos* spp. In addition, the biological characteristics of *Xenos* spp. were mostly consistent with previous studies conducted in Japan [1,15,16,17,30,31,32,33]. However, considering the wide distribution area of both *Vespa* spp. and *Xenos* spp., further studies of their biological interaction are necessary to be conducted in different localities, i.e., different climate conditions. Biological and genetic studies have intensively proceeded regarding the *P. dominula*–*X. vesparum* system [18,19,20]. There, detailed field observations and genomic/transcriptomic analysis have revealed the life cycle and mechanisms of their host manipulation [18,19,20,40,41].

Considering the high parasitism ratio and accessibility, the *V. analis*–*X. oxyodontes* system could be a good comparison of the *P. dominula*–*X. vesparum* system.

## Figures and Tables

**Table 1:** Number of collected and parasitized wasps and their rate of parasitism.

Host Species (Total Number)	Collection Method	Number of Wasps		
		Collected	Parasitized by *Sphaerularia vespae*	Parasitized by *Xenos* spp.
*Vespa mandarinia* (661)	Trap	181	1 (0.6)	6 (3.3)
	Net	480	2 (0.4)	7 (1.5)
*Vespa simillima* (303)	Trap	13	0 (0.0)	0 (0.0)
	Net	290	0 (0.0)	0 (0.0)
*Vespa analis* (457)	Trap	182	0 (0.0)	66 (36.3)
	Net	275	0 (0.0)	13 (4.7)
*Vespa dybowskii* (4)	Trap	3	0 (0.0)	0 (0.0)
	Net	2	0 (0.0)	0 (0.0)
*Vespa ducalis* (158)	Trap	62	1 (1.6)	2 (3.2)
	Net	96	0 (0.0)	0 (0.0)
*Vespa crabro* (57)	Trap	41	0 (0.0)	3 (7.3)
	Net	16	0 (0.0)	0 (0.0)

**Table 2:** Collection information of *Sphaerularia vespae*.

Host Species	Host Indiv. ID	Collection Method	Date
*Vespa mandarinia*	2021-0021	Trap	4 May
	2021-0069	Net	18 May
	2021-0122	Net	27 May
*Vespa ducalis*	2021-0137	Trap	31 May

**Table 3:** Variation and host of mitochondrial haplotypes of *Xenos* spp.

**(A): *Xenos moutoni***						
**Code** **(Number)**	**GenBank Accession Number**	**Host** **Species**	**Base Count from 5′ End in 652 bps Segment**			
			109	112	436	544
A (1)	LC764843	*Vespa mandarinia*	C	A	A	A
B (1)	LC764844	*Vespa mandarinia*	T	T	A	G
C (1)	LC764845	*Vespa mandarinia*	T	A	G	A
D (13)	LC764846	*Vespa mandarinia*, *V. ducalis*, *V. crabro*	T	A	A	G
**(B): *Xenos oxyodontes***						
**Code** **(Number)**	**GenBank Accession Number**	**Host** **Species**	**Base Count from 5′ End in 652 bps Segment**			
			286	340	391	463
E (1)	LC764847	*Vespa analis*	A	T	A	C
F (2)	LC764848	*Vespa analis*	G	C	A	C
G (19)	LC764849	*Vespa analis*	G	T	T	C
H (30)	LC764850	*Vespa analis*	G	T	A	A

**Table 4:** Average number and sex ratio of *Xenos* spp. parasitizing an infected host.

Host Species (Number of Host Individuals)	*Xenos* Species	Female	Male	Total	Sex Ratio
*Vespa mandarinia* (13)	*Xenos moutoni*	1	0	1	100
*Vespa analis* (77)	*Xenos oxyodontes*	0.82 ± 0.74 (0–5)	0.69 ± 0.98 (0–4)	1.51 ± 1.25 (1–9)	54.3
*Vespa ducalis* (2)	*Xenos moutoni*	1.5 ± 0.71 (1–2)	0	1.5 ± 0.71 (1–2)	100
*Vespa crabro* (3)	*Xenos moutoni*	0.33 ± 0.56 (0–1)	0.66 ± 0.56 (0–1)	1	33

**Table 5:** Seasonal variation in total number and sex ratio (% of female) of *Xenos* spp.

Species	Host	Sex	May	Jun.	Jul.	Aug.	Sept.	Oct.	Nov.	Total
*Xenos* *moutoni*	*Vespa mandarinia*, *V. ducalis*, *V. crabro*	Female	0	0	0	8	6	3	0	17
		Male	0	1	0	1	0	0	0	2
		Sex ratio	-	0	-	88	100	100	-	89
*Xenos* *oxyodontes*	*Vespa analis*	Female	6	17	11	24	4	1	0	63
		Male	0	0	11	27	11	2	2	53
		Sex ratio	100	100	50	47	27	33	0	54

*Xenos* parasitism was not detected in April, and thus, the table starts from May. *Xenos* parasitism was confirmed only in trapped hosts in *Vespa ducalis* and *V. crabro*. *Xenos moutoni* is significantly female-biased (*p* < 0.01).

**Table 6:** Summary of parasitism of *Xenos* spp. on the *Vespa* spp. captured by bait traps provided in the previous studies.

Host	Expected *Xenos* Species	Locality (Climate) *	Parasitism (%)	Reference
*Vespa mandarinia*	*Xenos moutoni*	Hokkaido (SA-CT)	0	[17]
		Niigata (CT)	16.7–23.5	[32]
		Ibaraki (T)	0	[30]
		Kyoto (T-WT)	3.3 (2.0)	This study
		Miyazaki (WT)	3.8	[16]
*Vespa ducalis*	*Xenos moutoni*	Hokkaido (SA-CT)	Not collected	[17]
		Niigata (CT)	12.5	[32]
		Ibaraki (T)	<2.0	[30]
		Kyoto (T-WT)	3.2 (1.3)	This study
		Miyazaki (WT)	3.8	[16]
*Vespa dybowskii*	*Xenos moutoni*	Hokkaido (SA-CT)	0	[17]
		Niigata (CT)	Not collected	[32]
		Ibaraki (T)	Not collected	[30]
		Kyoto (T-WT)	0 (0)	This study
		Miyazaki (WT)	Not collected	[16]
*Vespa crabro*	*Xenos moutoni*	Hokkaido (SA-CT)	<0.5	[17]
		Niigata (CT)	10.1	[32]
		Ibaraki (T)	<2.0	[30]
		Kyoto (T-WT)	7.3 (5.3)	This study
		Miyazaki (WT)	Not collected	[16]
*Vespa simillima*	*Xenos moutoni* or *X. oxyodontes*	Hokkaido (SA-CT)	<0.5	[17]
		Niigata (CT)	0.4–1.2	[32]
		Ibaraki (T)	<2.0	[30]
		Kyoto (T-WT)	0 (0)	This study
		Miyazaki (WT)	1.4	[16]
*Vespa analis*	*Xenos oxyodontes*	Hokkaido (SA-CT)	ca 8.0	[17]
		Niigata (CT)	8.8–16.7	[32]
		Ibaraki (T)	15–20	[30]
		Kyoto (T-WT)	36.3 (16.8)	This study
		Miyazaki (WT)	10.6	[16]

* The localities are ordered from north to south, and abbreviations for climate are as follows: CT = cool temperate; SA: subarctic; T: temperate; WT: warm temperate. A case of *X. moutoni* parasitism of *V. dybowskii* found in a hand-collected individual was noted in Sayama [17]. Parasitism ratio for the present study is written in a form ratio for bait-trapped hosts (total collected hosts).

## Data Availability

All original data are provided either in Supplementary Data S1 (collection information) or the GenBank database (molecular sequences determined in the present study).

## Supplementary Materials

The following supporting information can be downloaded at: https://www.mdpi.com/article/10.3390/insects14040398/s1, Table S1. PCR and sequencing primers used in the present study.; Supplementary Data S1. Collection information of *Vespa* spp.
