# Supplementary material for: Nematode and Strepsipteran Parasitism in Bait-Trapped and Hand-Collected Hornets (Hymenoptera, Vespidae, Vespa)
Source: Insects. 2023 Apr 20;14(4):398. doi: 10.3390/insects14040398 (PMC10143633; doi:10.3390/insects14040398)
Supplement: Supplementary file 1 [file insects-14-00398-s001.zip › Suppl. Table S1.pdf]

Supplementary Table S1. PCR and sequencing primers used in the present study.

Organism	Primer name	Sequence	Status
Sphaerularia vespae	SSU988F	CTC AAA GAT TAA GCC ATG C	1st and sequence PCR for anterior part of SSU
	SSU1912R	TTT ACG GTC AGA ACT AGG G	1st and sequence PCR for anterior part of SSU
	SSU1813F	CTG CGT GAG AGG TGA AAT	1st and sequence PCR for posterior part of SSU
	SSU2646R	GCT ACC TTG TTA CGA CTT TT	1st and sequence PCR for posterior part of SSU
	18SF	CGT AAC AAG GTA GCT GTA G	1st and sequence PCR for ITS
	D2aR	CAA CTT TCC CTC ACG GTA CTT GT	1st and sequence PCR for anterior part of LSU
	D1F	AAG GAT TCC CTT AGT AAC GGC GAT TG	1st and sequence PCR for LSU
	D4R	GCG GTA TTT GCT ACT ACC AYY AMG ATC TGC	1st and sequence PCR for LSU
	SSUR09	AGC TGG AAT TAC CGC GGC TG	Sequence PCR for anterior part of SSU (internal primer)
	SSUF22	TCC AAG GAA GGC AGC AGG C	Sequence PCR for anterior part of SSU (internal primer)
	SSUR13	GGG CAT CAC AGA CCT GTT A	Sequence PCR for posterior part of SSU (internal primer)
	SUF23	SATT CCG ATA ACG AGC GAG A	Sequence PCR for posterior part of SSU (internal primer)
	IKF1	GGG TCG ATG AAG AAC GCA G	Sequence PCR for ITS (internal primer)
	IKF2	CTG CGT TCT TCA TCG ACC	Sequence PCR for ITS (internal primer)
	D2a (	ACA AGT ACC GTG AGG GAA AGT TG	Sequence PCR for LSU (internal primer)
	D3a (	GAC CCG TCT TGA AAC ACG GA	Sequence PCR for LSU (internal primer)
	D3b	TCG GAA GGA ACC AGC	Sequence PCR for LSU (internal

		TAC TA	primer)
	D3aR	TCC GTG TTT CAA GAC GGG TC	Sequence PCR for LSU (internal primer)
Xenos spp.	LCO1490	GGT CAA CAA ATC ATA AAG ATA TTG G	1st and sequence PCR for *mtCOI*
	HCO2198	TAA ACT TCA GGG TGA CCA AAA AAT CA	1st and sequence PCR for *mtCOI*
